# Use of levosimendan combined with Shenfu injection to treat acute heart failure patients with hypotension: a prospective randomized controlled single-blind study

**DOI:** 10.1186/s12872-022-02572-2

**Published:** 2022-03-29

**Authors:** Miaomiao Li, Yue Zhang, Qianli Wan, Yiou Li, Tianzhen Qu, Fang Yuan

**Affiliations:** 1 Department of Critical Care Medicine (Specialty of Heart Failure), Tongren Hospital Shanghai Jiao Tong University School of Medicine, No. 1111 Xianxia Road, Shanghai, 200050 China; 2 Shanghai Jiao Tong University School of Medicine, Shanghai, 200025 China

**Keywords:** Acute decompensated heart failure, Brain natriuretic peptide, Cardiorenal rescue, Hemodynamic, Levosimendan, Shenfu injection

## Abstract

**Background:**

Levosimendan can improve clinical symptoms and the cardiorenal rescue success rate, and stabilize hemodynamic parameters in individuals suffering from acute decompensated heart failure. In addition, Shenfu injection (SFI) has been shown to protect the ischemic heart and enhance myocardial contractility.

**Methods:**

For this randomized control single-blind study, 101 patients with acute decompensated heart failure (ADHF) were enrolled and randomly assigned to control levosimendan (n = 51) and levosimendan + SFI injection (n = 50) groups. Attending physicians were not blinded for which arm the patients were allocated. Blood pressure, heart rate, the electrocardiogram, respiratory rate, fluid intake and urine output were all recorded 2 h and 24 h after drug infusions had commenced, and the cardiac index (CI) was monitored by ultrasonic cardiac output monitors.

**Results:**

Median blood pressure was markedly increased in the levosimendan + SFI group after 2 h and 24 h from the initiation of infusions compared to levosimendan administration alone. Brain natriuretic peptide (BNP) concentrations were reduced after administrations of levosimendan + SFI or solely levosimendan (both *P* < 0.001). Alterations in BNP concentrations were not different in the combination and control groups. No differences were found between the 2 groups in heart rate or severe hypotension, but blood pressure (systolic blood pressure, diastolic blood pressure) and hemodynamic parameters including CI, cardiac output and stroke volume index responded better in the levosimendan + SFI group compared to the monotherapy levosimendan group.

**Conclusions:**

Levosimendan + SFI was superior to treat ADHF patients compared to levosimendan monotherapy and produced significant improvements in hemodynamic parameters especially for ADHF patients with hypotension.

*Trail registration* The study was prospectively registered at Chinese Clinical Trial Registry with registration number [ChiCTR2000039385] (10/25/2020).

**Supplementary Information:**

The online version contains supplementary material available at 10.1186/s12872-022-02572-2.

## Background

Since 2000, levosimendan has been used to treat patients with acute decompensated heart failure (ADHF), because it produces safe hemodynamic stabilization [1]. Levosimendan promotes inotropy by sensitizing cardiac muscle troponin C (cTnC) to Ca^2+^ [2, 3]. In addition, it causes vasodilatation by activating ATP-dependent-K^+^ channels on smooth muscle. In a number of clinical trials, it was found that levosimendan elicited fewer adverse effects compared to other inotropic and vasoactive drugs; it is noteworthy that it still produced hypotension. One expert consensus report suggested the use of levosimendan in patients with pulmonary congestion. It is preferred to adrenoceptor agonists as first-line therapy for acute heart failure with acute coronary syndrome (ACS-AHF) patients on β-blocker medication, with unsatisfactory urine outputs after the administration of diuretics. It can be given as sole therapy or together with other inotropic or vasopressor drugs. However, patients should be closely monitored because of the risk of hypotension [4]. Lochner et al. [5] also reported that levosimendan combined with β-blockers or adrenergic inotropes did not inhibit the actions of levosimendan alone.

In patients with ADHF, levosimendan has been shown to significantly increase cardiac output (CO) and stroke and decrease pulmonary capillary wedge pressure (PCWP), mean blood, pulmonary artery and mean right atrial pressures, and total peripheral resistance [6]. For patients whose systolic blood pressure (SBP) is < 90 mmHg with hypoperfusion symptoms, it is necessary to raise CO, blood pressure and peripheral perfusion, to safeguard the functions of vital organs [7]. For acute heart failure (AHF) patients with low-output states, a drug that augments CO and increases the degree of vasodilatation would be expected to have better efficacy than one that augments CO alone.

Chemical analysis has shown that Shenfu injection mainly contains ginsenoside and higenamine [8, 9]. A Shenfu injection can increase arterial oxygen partial pressure and oxygen saturation [10, 11], increase hypoxia tolerance and anti-stress ability [12], reduce peripheral resistance and improve the microcirculation [13], increase myocardial contractility and cardiac output [14], dilate peripheral blood vessels [15], and improve the hypoxia-ischemic state of tissues and organs [16]. Ginsenosides can increase myocardial contractility, reduce small vascular resistance and increase cardiac, cerebral and renal perfusion [17]. Higenamine can not only improve myocardial contractility, but also dilate blood vessels and reduce cardiac load [18]. In the presence of ginsenosides, higenamine retains positive inotropic effects without positive frequency actions and thereby does not increase myocardial oxygen consumption [19]. In recent years, a number of clinical trials have shown that the Chinese patent medicine, Shenfu injection (SFI), greatly improved the symptoms of heart failure (HF) [7–9]. The mechanisms involved in the actions of SFI include a significant reduction in taurine, glutathione and phospholipids concentrations. This was shown in an ischemic heart failure rat model, when the distribution of these molecules in the non-infarct zone was markedly altered [20]. In clinical trials, SFI not only improved CO but also the vasodilator dimension [14, 21].

Therefore, in the present study, we hypothesized that levosimendan combined with SFI would improve cardiac functions without producing hypotension and improve the symptoms of patients with both ADHF and hypotension.

## Methods

The study involved a cohort of 101 patients suffering from ADHF from 2019.12 to 2021.6 They were diagnosed according to the 2018 Chinese guidelines [22] and randomly assigned into control (levosimendan + placebo) (n = 51) and study (levosimendan + SFI) (n = 50) groups. Inclusion criteria were based on the New York Heart Association grading guidelines [22] and were: patients had grade III or IV; an left ventricular ejection fraction (LVEF) ≤ 40%; and a brain natriuretic peptide (BNP) level > 400 pg/mL. Some patients were also diagnosed with low cardiac output syndrome (LCOS). All enrolled patients were not allergic to traditional Chinese herbal medicines.

The study followed the Declaration of Helsinki principles and was approved by the Institutional Review Board of Tongren Hospital affiliated to Shanghai Jiao Tong University School of Medicine. All enrolled patients provided informed consent. The registered trial number was ChiCTR2000039385.

The exclusion criteria for patients were: of childbearing potential; HF due to restrictive or hypertrophic cardiomyopathy or stenotic valvular disease that was uncorrected; had acute myocardial infarction 14 days prior to the study or had refractory angina; sustained ventricular arrhythmia; severe liver and/or kidney insufficiency; severe infection; malignant tumor; systemic immune disease; those who would not cooperate with treatment; withdrawal from the study; or death.

### Study procedure

The enrolled patients were randomized into 2 groups using randomization numbers generated by SPSS software. One group received a levosimendan infusion of 12 µg/kg in 0.9% sodium chloride and a placebo (5% GS 350 mL) and the other group received the same levosimendan infusion plus SFI (100 mL + 5% GS 250 mL). There were digital labels coded 1–100, enrolled patients blindly extracted labels, the patients with odd number were assigned into the levosimendan group, while patients with even numbers were assigned into the levosimendan + SFI group. The labels were discarded after each extraction, and newly enrolled patients extracted from the remaining labels, but attending physicians were not blinded for which arm the patient was allocated. This study enrolled 101 patients, the last patient extracted a label from the new digital labels which were coded 1–100 again.

Constant rate infusion of levosimendan was maintained for 24 h, unless the patient had a serious cardiovascular event, dose-limiting adverse events (AEs) or serious adverse events (SAEs), or required i.v. inotropic or vasodilator agents as rescue therapy. Standard clinical parameters included the electrocardiogram, blood pressure, heart and respiratory rates, fluid intake, output of urine were measured and recorded after 24 h infusion. The cardiac index (CI) was measured using ultrasonic cardiac output monitors (USCOM), which is a non-invasive CO monitor that employs transaortic or transpulmonary doppler flow tracing and valve area estimated using patient height to determine CO. The probe of USCOM was placed in the sternum or supraclavicular fossa to obtain the strongest signal. Three consecutive measurements were made with a deviation of no more than 10% each time, and the average CI was taken.

### Assessments

Patients were evaluated at baseline (before treatment) and during treatment for variables including their medical histories, physical examinations, echocardiography and laboratory blood tests. The concentrations of BNP in plasma were measured again at 24 h after initiation of drug administration. AEs were evaluated and recorded by clinicians for 24 h.

### Endpoints

The primary endpoint was the change in blood pressure (incidence of significant hypotension) when treatment was clinically effective 2 and 24 h after initiation of drug administration. For each measured variable, improvement was defined as a reduction in ≥ 1 grade from the baseline value.

Secondary endpoints included a decrease in the serum concentration of BNP from baseline and 24 h after the start of the infusion. In addition, heart rate (HR), CO, CI, stroke volume index (SVI) and systemic vascular resistance index (SVRI) parameters were evaluated 2 h and 24 h after initiation of drug administration and compared to baseline values.

### Statistical analysis

SAS ver. 9.2 was utilized for all estimations of sample sizes and analyses. Normally distributed continuous variables were analyzed using Student’s t-test or ANOVA with the Kruskal–Wallis test for significant differences between them and expressed as mean ± SD, while abnormally distributed continuous variables were analyzed using the Mann–Whitney U test or the Wilcoxon rank sum test and provided as median with interquartile range [IQR]). A χ^2^ or Fisher’s exact test was employed to look for differences between categorical variables. A *P* value < 0.05 was deemed to be a significant finding.

## Results

### Clinical characteristics of patients and baseline values

A total of 101 patients with AHF were screened between 2019.12 and January 2021.6 having met the inclusion criteria (vide supra). The median age (IQR) of the levosimendan group was 73 (67.00, 80.00) years and for the levosimendan combined SFI group 73 (69.00, 80.00) years. The general demographic characteristics and baseline data of the two groups were comparable with no significant differences between most parameters. Hemoglobin values were somewhat less in the SFI group, but still within the physiological range. Patients in both groups had hypotension and diabetes comorbidities and received various drugs including angiotensin system inhibitors, β-blockers and diuretics including spironolactone and torsemide before enrollment; it is noteworthy that > 90% of patients were taking β-blockers. Torsemide doses were higher in the solely levosimendan group (Table 1).

**Table 1:** Characteristics and baseline values of patients

	Levosimendan (N = 51)	Levosimendan + SFI (N = 50)	*P* value
Gender n (%) Female	15 (29.40)	11 (22.00)	0.394
Male	36 (70.60)	39 (78.00)	
Age (yr), median (IQR)	73.00 (67.00, 80.00)	73.00 (69.00, 80.00)	0.618
BMI (kg/m^2^), median (IQR)	23.89 (22.04, 25.39)	22.59 (20.43, 25.10)	0.108
Hemoglobin (g/L), mean ± SD	132.39 ± 18.34	123.76 ± 20.87	0.030
Creatinine (μmoI/L), median (IQR)	94.10 (76.30, 116.60)	104.20 (83.10, 118.70)	0.443
eGFR (mL/min/1.73 m^2^), median (IQR)	68.00 (57.40, 83.87)	59.78 (52.43, 82.57)	0.237
LVEF (%), median (IQR)	38.00 (33.30, 39.00)	38.00 (32.00, 39.00)	0.624
BNP (ng/L), median (IQR)	1169 (482.42, 2168.33)	1415.81 (501.80, 3056.70)	0.220
Hypotension, n (%)	37 (72.55)	38 (76.00)	0.692
Etiology of heart failure, n (%)			0.364
Hypertrophic cardiomyopathy	1	0	
Alcoholic cardiomyopathy	0	1	
Hypertensive heart disease	1	2	
Coronary heart disease	36	38	
Dilated cardiomyopathy	10	4	
Valvular heart disease	3	5	
Previous admission with heart failure, n (%)	44 (86.27)	44 (88.00)	1.000
Comorbidities, n (%)			
Hypertension	37 (72.55)	41 (82.00)	0.344
Diabetes mellitus	26 (50.98)	29 (58.00)	0.479
Cerebral stroke	5 (9.80)	7 (14.00)	0.554
COPD	3 (5.88)	6 (12.00)	0.318
Hyperlipidemia	5 (9.80)	7 (14.00)	0.554
Pre-admission medication			
ACEI/ARB/ARNI, n (%)	45 (88.24)	43 (86.00)	0.775
Sacubitril/Valsartan	43 (84.31)	43 (86.00)	
Valsartan	1 (1.96)	0	
Enalapril	1 (1.96)	0	
β-blocker, n (%)	46 (90.20)	49 (98.00)	0.205
Metoprolol	43 (84.31)	44 (88.00)	
Bisoprolol	2 (3.90)	1 (2.00)	
Carvedilol	1 (1.96)	4 (2.00)	
Spironolactone, n (%)	42 (82.35)	40 (80.00)	0.762
Loop diuretic	37 (72.55)	39 (78.00)	0.141
Torsemide	32 (62.75)	39 (78.00)	
Furosemide	5 (9.80)	0	
Pre-admission medication dose			
ACEI/ARB/ARNI			
Sacubitril/Valsartan (mg, bid), median (IQR)	50 (50, 50) [n = 43]	50 (25, 50) [n = 43]	0.303
Valsartan (mg, qd)	80 [n = 1]	– [n = 0]	–
Enalapril (mg, qd)	10 [n = 1]	– [n = 0]	–
β-blocker			
Metoprolol (mg, qd), median (IQR)	47.50 (23.75, 47.50) [n = 43]	47.50 (23.75, 47.50) [n = 44]	> 0.999
Bisoprolol (mg, qd)	5 [n = 2]	5 [n = 1]	–
Carvedilol (mg, bid), median	10 [n = 1]	10 [n = 4]	–
Spironolactone (mg, qd)	20 [n = 42]	20 [n = 40]	–
Loop diuretic			
Torsemide (mg, qd), median (IQR)	20 (20, 20) [n = 32]	10 (10, 10) [n = 39]	< 0.001
Furosemide (mg, qd)	20 [n = 5]	– [n = 0]	–

*ACEI* angiotensin-converting enzyme inhibitors, *ARB* angiotensin-receptor blocker, *ARNI* angiotensin receptor neprilysin inhibitor, *BMI* body mass index, *BNP* brain natriuretic peptide, *COPD* chronic obstructive pulmonary disease, *eGFR* estimated glomerular filtration rate, *LVEF* left ventricular ejection fraction

Medications during admission are listed in Additional file 1: Table 1. There was no significant difference between the 2 groups.

### Endpoints

#### Primary endpoint (clinical effects)

**Fig. 1:**
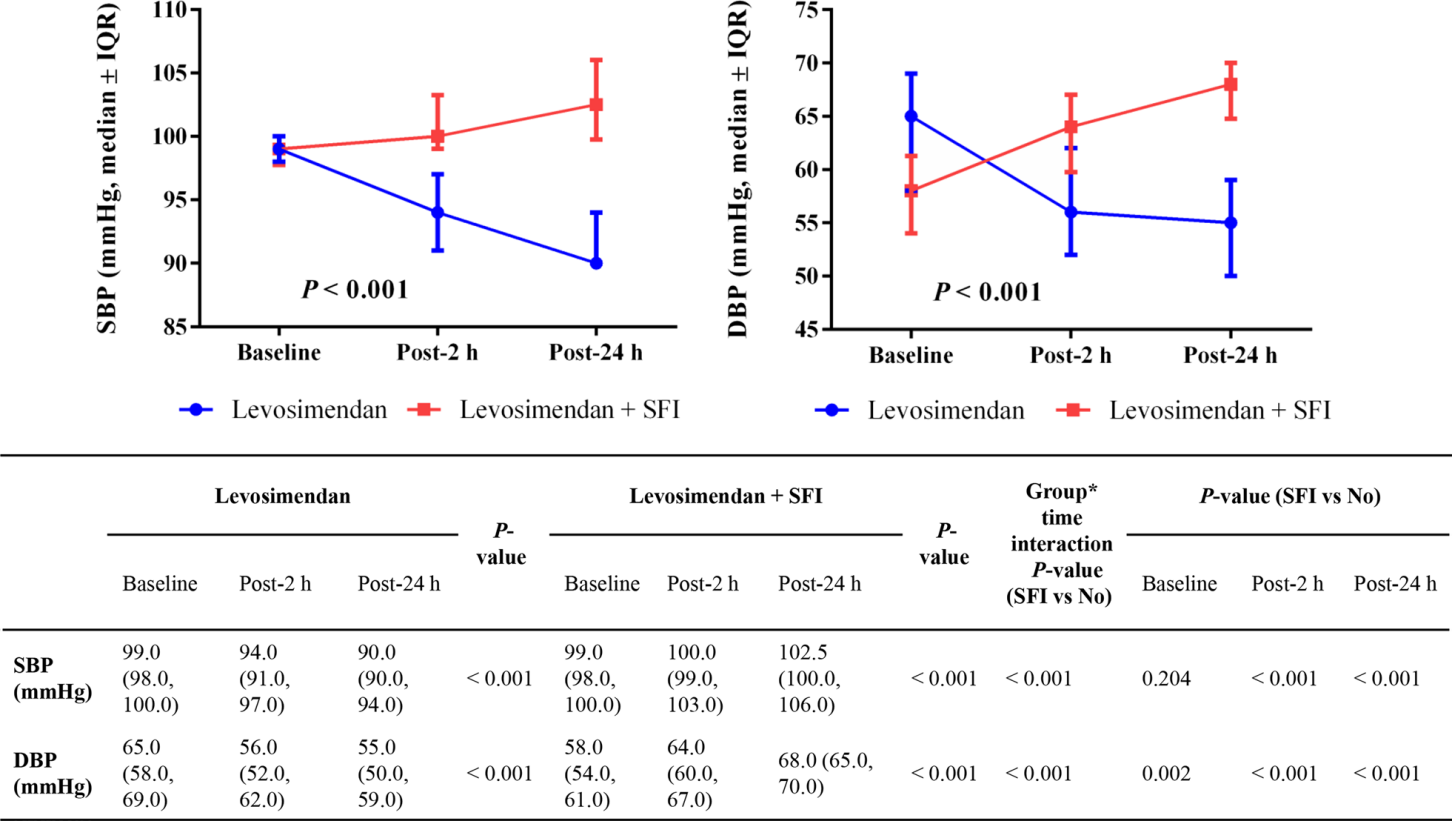
The change in blood pressure 2 h and 24 h after initiation of drug administration. *Note*: All data are shown as median (IQR). Abbreviations: *DBP* diastolic blood pressure, *SBP* systolic blood pressure

The blood pressure including SBP and diastolic blood pressure (DBP) after SFI combined with levosimendan were significantly increased at different time points, but were still within the normal range [median: 102.5 (IQR: 100.0, 106.0)/median: 68.0 (IQR: 65.0, 70.0)]. The SBP and DBP of levosimendan were significantly decreased [median: 90.0 (IQR: 90.0, 94.0)/median: 55.0 (IQR: 50.0, 59.0)] 24 h after the infusion initiation (Fig. 1).

### Secondary endpoints

**Fig. 2:**
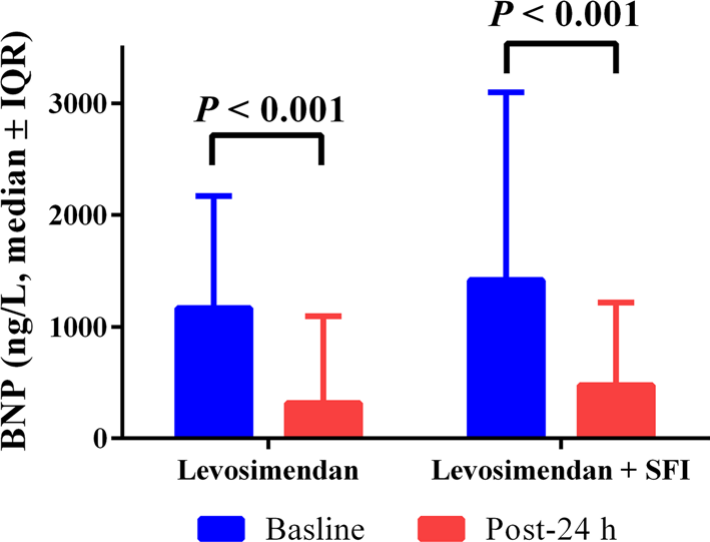
Changes in BNP before and after therapy. *Note*: All data are shown as median (IQR). Abbreviations: *BNP* brain natriuretic peptide, *SFI* Shenfu injection

The BNP values in both groups significantly decreased compared to baseline values, the change in the concentrations of BNP were not different in the combination and control groups (Fig. 2).

In addition, with regard to heart rate (HR) there were no significant differences between the two groups. Hemodynamic parameters including CI, CO and SVI were superior improved in the levosimendan combined with SFI group than in the levosimendan monotherapy group. Similarly, although the SVRI at 24 h appeared to be different in both groups compared to baseline, but statistical significance was not reached (*P* = 0.076) for the group differences over time.

**Fig. 3:**
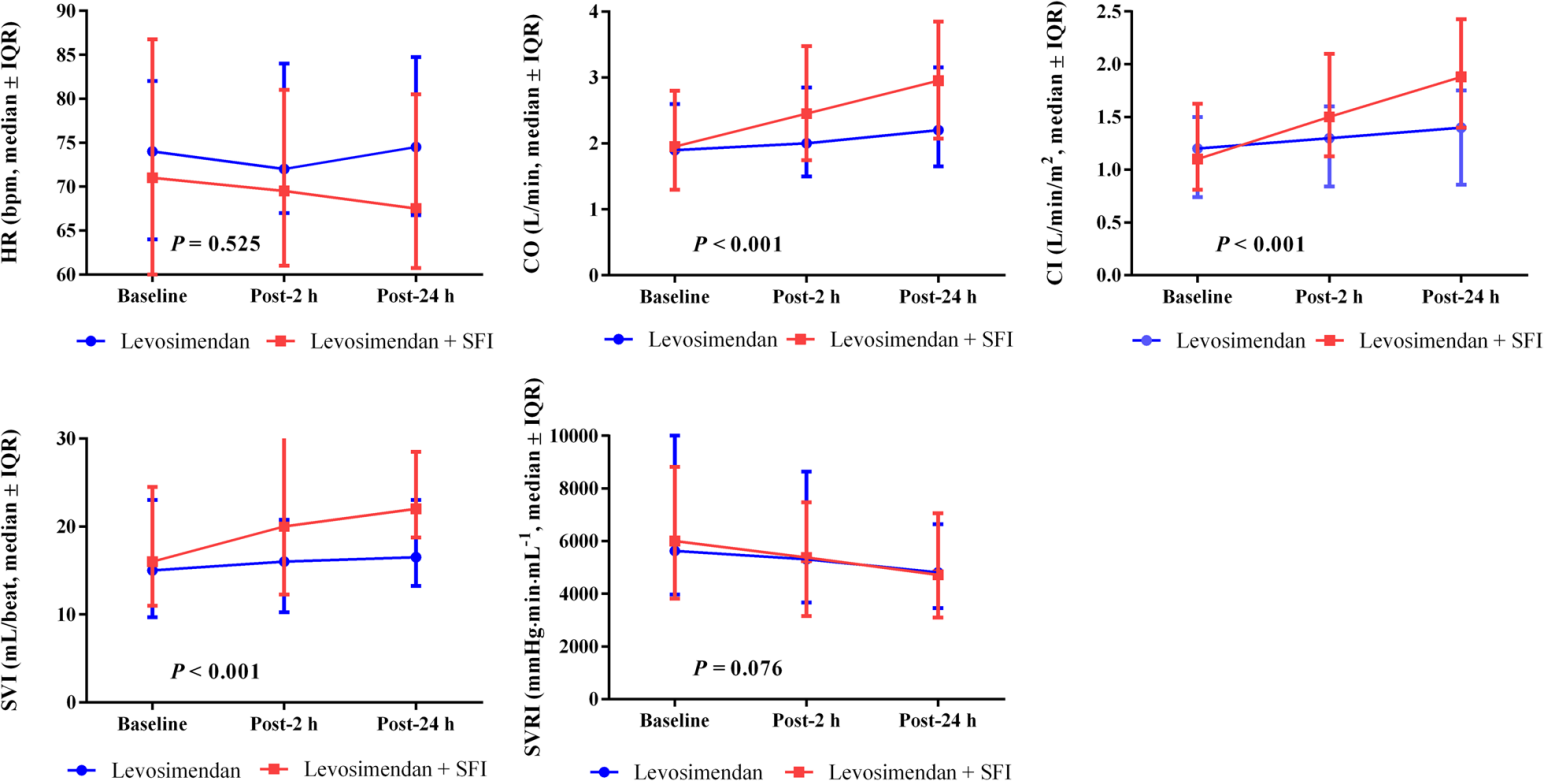
Comparison of the differences in the changes of hemodynamic parameters over time between the two groups (groups * time interaction). Abbreviations: *CI* cardiac index, *CO* cardiac output, *HR* heart rate, *SFI* Shenfu injection, *SVI* stroke volume index, *SVRI* systemic vascular resistance index

The differences in the changes of CO, CI and SVI hemodynamic parameters over time were all significantly enhanced in the levosimendan combined with SFI group (*P* < 0.05) (Fig. 3, Table 2).

**Table 2:** Comparison of hemodynamic parameters of the patients between levosimendan combined with Shenfu injection and levosimendan alone

	Levosimendan			*P* value	Levosimendan + SFI			*P* value	*P* value (SFI *vs* No)	*P* value (SFI vs No)		
	Baseline	Post-2 h	Post-24 h		Baseline	Post-2 h	Post-24 h			Baseline	Post-2 h	Post-24 h
HR (bpm)	74.0 (64.0, 82.0)	72.0 (67.0, 84.0)	74.5 (67.0, 84.0)	0.833	71.0 (60.0, 86.0)	69.5 (61.0, 81.0)	67.5 (61.0, 80.0)	0.927	0.525	0.265	0.210	0.042
CO (L/min)	1.9 (1.3, 2.6)	2.0 (1.5, 2.8)	2.2 (1.7, 3.0)	0.420	1.95 (1.3, 2.8)	2.45 (1.77, 3.45)	2.95 (2.1, 3.8)	0.001	< 0.001	0.676	0.084	0.019
CI (L/min/m^2^)	1.2 (0.74, 1.5)	1.3 (0.85, 1.5)	1.4 (0.86, 1.7)	0.347	1.1 (0.82, 1.6)	1.5 (1.15, 2.1)	1.88 (1.4, 2.4)	< 0.001	< 0.001	0.889	0.009	0.003
SVI (mL/beat)	15.0 (9.7, 23.0)	16.0 (10.5, 20.5)	16.5 (13.5, 23.0)	0.516	16.0 (11.0, 24.0)	20.0 (12.5, 30.5)	22.0 (19.0, 28.0)	0.004	< 0.001	0.501	0.024	0.003
SVRI (mmHg·min·mL^−1^)	5633.0 (3969.0, 10,000.0)	5315.0 (3773.0, 8274.0)	4816.0 (3591.0, 6217.0)	0.214	6001.5 (3895.0, 8817.0)	5379.0 (3200.0, 7454.0)	4722.5 (3141.0, 6867.0)	0.250	0.076	0.763	0.320	0.705

All data are shown as median (IQR)

*CO* cardiac output, *CI* cardiac index, *SVI* stroke volume index, *SVRI* systemic vascular resistance index

It became also evident from inspection of Table 3 that the changes elicited by levosimendan combined with SFI and levosimendan alone mainly improved cardiac function, and therefore blood pressure. It was more beneficial for patients with AHF and hypotension included in the present study.

**Table 3:** Comparison the difference of changes in hemodynamic parameters of patients from post 24 h to baseline between levosimendan combined with Shenfu injection and levosimendan alone groups

	Levosimendan	Levosimendan + SFI	*P* value
	Changes of post 24 h-baseline	Changes of post 24 h-baseline	
BNP (ng/L)	− 908.68 (− 1849.92, − 446.57)	− 932.64 (− 1906.30, − 247.74)	0.741
SBP (mmHg)	− 8.00 (− 10.00, − 4.00)	4.00 (2.00, 9.00)	< 0.001
DBP (mmHg)	− 9.00 (− 12.00, − 4.00)	9.00 (6.00, 12.00)	< 0.001
HR (bpm)	0.50 (− 4.00, 7.00)	0.50 (− 6.00, 7.00)	0.525
CO (L/min)	0.10 (− 0.20, 0.50)	0.70 (0.50, 1.20)	< 0.001
CI (L/min/m^2^)	0.10 (− 0.10, 0.40)	0.61 (0.21, 0.80)	< 0.001
SVI (mL/beat)	0.09 (− 2.00, 3.40)	6.00 (3.00, 9.00)	< 0.001
SVRI (mmHg·min·mL^−1^)	− 862.00 (− 2704.00, 51.00)	− 494.50 (− 2249.00, 780.00)	0.076

All the data are shown as median (IQR)

*BNP* brain natriuretic peptide, *CI* cardiac index, *CO* cardiac output, *DBP* diastolic blood pressure, *HR* heart rate, *SBP* systolic blood pressure, *SFI* Shenfu injection, *SVI* stroke volume index, *SVRI* systemic vascular resistance index

### Kidney and liver functions

Table 4 shows that there were no significant differences in serum creatinine (Scr), blood urea nitrogen (BUN), alanine transaminase (ALT) and aspartate aminotransferase (AST), respectively between the 2 groups over 24 h (all *P* > 0.05).

**Table 4:** Kidney and liver function indicators in the two groups

	Levosimendan	Levosimendan + SFI	*P* value
	Changes in post 24 h-baseline	Changes in post 24 h-baseline	
Scr (μmoI/L)	3.45 (− 2.95, 21.25)	5.30 (− 8.00, 23.50)	0.752
BUN (mmol/L)	0.60 (− 1.73, 2.40)	− 0.04 (− 3.41, 3.45)	0.251
ALT (U/L)	− 2.50 (− 7.50, 4.50)	− 2.00 (− 10.00, 7.00)	0.788
AST (U/L)	0.00 (− 10.50, 10.00)	0.00 (− 19.00, 5.00)	0.363

All data are shown as median (IQR)

*ALT* alanine transaminase, *AST* aspartate aminotransferase, *BUN* blood urea nitrogen, *Scr* serum creatinine, *SFI* Shenfu injection

## Discussion

In the present study, we confirmed that levosimendan combined with SFI effectively increased blood pressure, which was reduced in patients with AHF due to insufficient peripheral blood volume. AHF refers to an attack or aggravation of the functions of the left heart, mainly due to reduced myocardial contractility, an increase in cardiac load, and pressure in the pulmonary circulation, and raised resistance of the peripheral circulation. Pulmonary congestion and edema, together with poor organ perfusion and cardiogenic shock are the most common clinical syndromes caused by pulmonary circulation congestion [23, 24]. Therefore, AHF has a relatively high in-hospital mortality rate of 3%, and 3- and 5-year mortality rates of 30% and 60%. The pathogenesis of AHF is complex, but most studies have demonstrated that it is related to hemodynamic disorders [25, 26].

The results of epidemiological investigation have shown that the incidence of AHF has been increasing in recent years in China, and has seriously affected people's physical and mental health and their quality of life [27]. Our results strongly suggest that the addition of an adjuvant significantly improved the hemodynamic indicators CO, CI, SVI and SVRI, which will naturally improve the survival rate and prognosis of patients.

Both levosimendan and SFI are relatively common drugs used to treat HF in China. Among them, levosimendan is a positive inotropic drug, which can bind with troponin C after drug action, increasing the sensitivity of contractile protein to Ca^2+^, thus improving myocardial contractility and reducing cardiac load. However, the effect of this drug alone is not ideal [28] for ADHF patients with hypotension. We also use SFI in clinical practice. Ginsenosides and aconitine alkaloids in SFI are the main active components [29]. Ginsenoside in red ginseng can reduce myocardial oxygen consumption and enhance myocardial contractility, while normethylidene alkaloid in aconitine alkaloids, has the effect of anti-myocardial ischemia and heart strengthening [30].

From ex vivo experiments in a septic shock rabbit model, we found that SFI could increase mean arterial pressure (MAP), decrease serum lipopolysaccharide (LPS), lactate dehydrogenase (LDH) and AST concentrations, and improve the tissue morphology of the heart, liver and kidney. In addition, SFI can re-increase the concentrations of ATP and taurine while reducing the concentration of AMP in cardiac muscle during septic shock [29].

Much research has been carried out on cardiac functions in patients with ADHF who have been treated with levosimendan or levosimendan combination drugs. Many studies have confirmed that levosimendan can significantly improve CO, reduce the BNP concentration and increase LVEF in patients with ADHF [31, 32]. In the present study, it was clear that similar effects of levosimendan combined with SFI and levosimendan therapies decreased the BNP concentration, although addition of SFI did not significantly improve the heart rate.

Levosimendan-nesiritide combination therapy produced the most pronounced improvements during the early stages of treatment, which gradually declined to the same levels produced by monotherapies at day 9 [33]. Therefore, for patients with ADHF, combination therapies achieved clinical efficacies faster than respective monotherapies, but improvements in the long term may well be similar.

Levosimendan and SFI have different mechanisms of action; levosimendan is a positive inotropic drug which does not raise the free intracellular Ca^2+^ concentration. In theory, a combination of these two drugs should produce synergistic effects greater than those produced by administration of only one of the drugs.

## Study limitations

The results of this study should not be regarded as definitive regarding whether an SFI infusion affected BNP concentrations. Although BNP has a brief half-life, there was a delay of up to 48 h from cessation of drug administration to the determination of the BNP concentration. Due to the lack of measurements of proANP and aldosterone in laboratory tests, the BNP concentrations were only assessed 24 h after drug administration. In an ideal world, however, it would be desirable if all the concentrations (BNP, proBNP and aldosterone) were included. In addition, it is possible that a single-center study may introduce bias. Our study had a relatively small sample size, which would weaken the primary endpoint. Larger clinical trials are needed to confirm the improvement in hemodynamic parameters of SFI combined with levosimendan. Furthermore, SFI may not be available outside China, which leads to an issue of generalizability.

## Conclusion

Intravenous infusion of levosimendan and SFI to acute decompensated heart failure patients with hypotension was a superior treatment compared to levosimendan monotherapy with regard to hemodynamic parameters.

## Supplementary Information

**Additional file 1:** Clinical study protocol.

**Additional file 2: Supplementary Table 1.** Medications used during the admission.

## Data Availability

The datasets used and/or analyzed during the current study are available from the corresponding author on reasonable request.

## Abbreviations

ACS-AHF: Acute heart failure with acute coronary syndrome
ADHF: Acute decompensated heart failure
AEs: Adverse events
AHF: Acute heart failure
ALT: Alanine transaminase
AST: Aspartate aminotransferase
BNP: Brain natriuretic peptide
BUN: Blood urea nitrogen
CI: Cardiac index
cTnC: Cardiac muscle troponin C
CO: Cardiac output
DBP: Diastolic blood pressure
HF: Heart failure
HR: Heart rate
LCOS: Low cardiac output syndrome
LDH: Lactate dehydrogenase
LVEF: Left ventricular ejection fraction
LPS: Lipopolysaccharide
MAP: Mean arterial pressure
PCWP: Pulmonary capillary wedge pressure
SBP: Systolic blood pressure
SAEs: Serious adverse events
Scr: Serum creatinine
SFI: Shenfu injection
SVI: Stroke volume index
SVRI: Systemic vascular resistance index
USCOM: Ultrasonic cardiac output monitors
