# Supplementary material for: Use of levosimendan combined with Shenfu injection to treat acute heart failure patients with hypotension: a prospective randomized controlled single-blind study
Source: BMC Cardiovasc Disord. 2022 Mar 29;22:130. doi: 10.1186/s12872-022-02572-2 (PMC8966164; doi:10.1186/s12872-022-02572-2)
Supplement: Supplementary file 1 — Additional file 1: Clinical study protocol. [file 12872_2022_2572_MOESM1_ESM.docx]

**Clinical study protocol**

Efficacy and safety of Levosimendan combined with Shenfu injection in acute heart failure with hypotension

Scheme version No.: V 1.0

Version Date: 2020.0.08.01

Principal investigator: Yuan Fang, director of Critical Care Medicine (heart failure specialty)

Applicants: Shanghai Tongren Hospital

# Summary of clinical trials

Study title: Efficacy and safety of Levosimendan with Shenfu injection for acute heart failure with hypotension

Applicants: Shanghai Tongren Hospital

Indications: Acute heart failure combined with hypotension

Objective: To evaluate the efficacy and safety of Levosimendan combined with Shenfu in patients with acute HF and hypotension.

Study design: This clinical trial adopts a single-center, prospective, randomized-controlled trial design. Patients who met acute heart failure with hypotension were randomized to treatment or control groups in a 1:1 ratio based on receiving optimized standard treatment for heart failure:

1. Test group: standard treatment + Levosimendan + NS injection;
2. Control group: standard treatment + Levosimendan + Shenfu injection.

Follow-up was performed for 5 days after the end of 24 hours of administration for end point events within 6 days of randomized initiation

Study population: NYNA grade III-IV acute heart failure patients with hypotension.

Selection criteria: Inclusion criteria were based on the New York Heart Association grading guidelines (14) and were as follows: patients had grade III or IV; an LVEF ≤ 40%; and a BNP level > 400 pg/mL. Traditional Chinese medicine (TCM) treatment was carried out following the clinical study of new Chinese medicines guidelines (the Golden mirror of medicine). Some patients were also diagnosed with asthenic heart syndrome. All enrolled patients were not allergic to traditional Chinese herbal medicines.

Exclusion criteria: patients of childbearing potential; HF due to restrictive or hypertrophic cardiomyopathy or stenotic valvular disease that was uncorrected; had acute myocardial infarction 14 days prior to the study or had refractory angina; sustained ventricular arrhythmia;severe liver and/or kidney insufficiency; severe infection; malignant tumor; systemic immune disease; those who would not cooperate with treatment; withdrawal from the study; or death.

art valvular disease; (3) Lack of treatment, withdrawal or death.

Efficacy evaluation indicators: (1) Hemodynamic improvement effect: USCOM monitoring of CI, CO, SVI, SVRI; (2) the reduction effect of BNP.

Safety assessment: blood pressure, heart rate and breathing changes before and after (1) Treatment; (2) Blood routine, liver and renal function, and electrolyte changes before and after treatment. (3) Record and assess any adverse event occurring and its association with the study drug.

Sample size: 100 planned patients with acute heart failure combined with hypotension.

1. Test background and principle

Acute heart failure (AHF) refers to the acute attack or aggravated abnormal myocardial contractility of left heart function, heart load, causing acute cardiac output, pulmonary circulation pressure, peripheral circulation resistance, cause pulmonary congestion and acute pulmonary congestion, pulmonary edema and can be accompanied by insufficient tissue, organ perfusion and cardiogenic shock clinical syndrome, with left heart failure is the most common. AHF is critically ill and has a poor prognosis. Relevant data show that the hospital case fatality rate of AHF is 3%, and the 3-and 5-year mortality rate reaches 30% and 60%, respectively, with poor prognosis and high case fatality rate. The pathogenesis of AHF is complex, but most scholars are associated with hemodynamic disorders.

Domestic and foreign heart failure guidelines recommended the use of positive muscle strength drugs for patients with AHF with hypotension (systolic blood pressure <90mmHg) and / or low perfusion in tissues and organs. Short-term intravenous application of positive muscle strength drugs can increase cardiac output, increase blood pressure, relieve tissue hypoperfusion, and maintain the function of vital organs. Traditional positive muscle strength drugs include dopamine, dobutamine and phosphodiesterase inhibitors. The first two have positive muscle strength through excited β1 receptor. Patients applying receptor blockers are not recommended and have side effects of increasing myocardial oxygen consumption; phosphodiesterase inhibitors have side effects of lower blood pressure and arrhythmias. Levosimendan is a new calcium ion sensitizer that produces positive muscle force with myocardial troponin C; open K channel of vascular smooth muscle cell membrane ATP sensitivity, expand peripheral blood tube, reduce before and posterior myocardial load; expand pulmonary artery, reduce pressure of pulmonary artery; expand coronary artery and improve blood supply to the myocardium. Levosimendan has good clinical efficacy in treating AHF. Levosimendan has a dual mechanism of action of positive muscle strength and vasodilation, so it has been found that early use can cause a decrease in cardiac output, mean pulmonary artery pressure, pulmonary capillary wedge pressure, and systemic vascular resistance. The incidence of hypotension in the LIDO study was 8.7%; the incidence of hypotension in the RUSSLAN study was 4 – 7% (0.1-0.2 μg / kg/min) and 9% (0.4 μg / kg/min), and the incidence of hypotension in the SURVIVE study was 15.5%.Clinically, many AHF patients with hypotension and low perfusion are prone to further lower blood pressure in the early stage, and have to reduce the speed of intravenous infusion, or even directly stop the drug, affecting the rescue of Levosimendan for AHF. Early use of side effects of hypotension is currently commonly used booster drugs are dopamine, dopamine butamine, these traditional positive muscle strength drugs can lead to arrhythmias, increase myocardial oxygen consumption, and thus increase patient mortality.

For AHF patients with low-output states, a drug that augments cardiac output and increases the degree of vasodilatation would be expected to have better efficacy than an one that augments cardiac output alone. In recent years, a number of clinical trials have shown that the Chinese patent medicine, shenfu injection (SFI), greatly improved the symptoms of HF. The mechanisms involved in the actions of SFI include a significant reduction in taurine, glutathione and phospholipids concentrations. This was shown in an ischemic heart failure rat model, when the distribution of these molecules in the non-infarct zone was markedly altered (13). In clinical trials, SFI not only improved cardiac output but also the VS vasodilator dimension.

Both the Shenfu Injection and Levosimendan are relatively commonly used therapeutic drugs, while Levosimendan is a new positive muscle strength drug, which can relieve clinical symptoms. Due to its vascular expansion effect, it is easy to lead to hypotension in the early stage. For patients with low discharge, its clinical application is limited. The Fuzi and Ginseng in the Shenfu Injection have a booster effect to counteract the hypotension and response caused by Levosimendan, benefit more patients with AHF combined with hypotension, and finally further improve cardiac function and improve the clinical treatment effect.

This study aimed to reduce the side effects of the BP reduction in early clinical application of levosimendan in patients with AHF with Shenfu Injection, which produced better hemodynamic effects.

Levosimendan and the participating injection of SFI in patients with hypotension overcame the side effects of accelerated heart rate and increased myocardial oxygen consumption of traditional booster drugs, and the two played a synergistic role in improving the hemodynamic effect of patients with AHF. The USCOM non-invasive hemodynamic evaluation method is simple, real-time, convenient, and accurate, and is capable of continuously monitoring HR, BP, CO, CI, SVI, and SVRI.

2. Test objective

The purpose of this study was to evaluate the role of improving hemodynamics by comparing Levosimendan alone with SFI with BNP levels as a measure of efficacy in 100 patients with standard treated acute HF with hypotension.

3. Research design

3.1. Summary

This clinical trial uses a randomized-controlled trial design. Patients who met the diagnosis of acute heart failure combined with hypotension were randomized to the trial or control groups in a 1:1 ratio on the basis of receiving the optimized standard heart failure treatment:

1) Test group: standard treatment + Levosimendan + Shenfu Injection; Levosimendan pump for 24 h, no load, dose 0.1-0.2 g / kg/min, dose adjusted according to blood pressure; Shenfu Injection 80ml + 5%GS 250ml.

2) Control group: standard treatment + Levosimendan + 0.9% NS injection; Levosimendan pumped for 24 h, without load, dose 0.1-0.2 g / kg/min, dose adjusted according to blood pressure, and 0.9% NS 80ml + 5%GS 250ml.

Follow-up was performed for 5 days after drug administration, and changes in efficacy and safety indicators were observed within 6 days after randomization.

3.2. Study population

Patients clearly diagnosed with acute heart failure combined with hypotension during 2020.09-2022.01 in our hospital.

Selection criteria: Inclusion criteria were based on the New York Heart Association grading guidelines (14) and were as follows: patients had grade III or IV; an LVEF ≤ 40%; and a BNP level > 400 pg/mL. Traditional Chinese medicine (TCM) treatment was carried out following the clinical study of new Chinese medicines guidelines ( the Golden mirror of medicine). Some patients were also diagnosed with asthenic heart syndrome. All enrolled patients were not allergic to traditional Chinese herbal medicines.

Exclusion criteria: patients of childbearing potential; HF due to restrictive or hypertrophic cardiomyopathy or stenotic valvular disease that was uncorrected; had acute myocardial infarction 14 days prior to the study or had refractory angina; sustained ventricular arrhythmia; severe liver and/or kidney insufficiency; severe infection; malignant tumor; systemic immune disease; those who would not cooperate with treatment; withdrawal from the study; or death.

3.3. Therapeutic regimen

The enrolled patients were randomized into 2 groups using randomization numbers generated by SPSS software. One group received an levosimendan injection of 12 µg/kg and 0.9% sodium chloride for 2 h and 24 h and the other group levosimendan (12.5 mg 0.9% NS 50 mL) plus an SFI (100 mL + 5% GS 250 mL).

Concomitant medication used: 1) Standard treatment for heart failure should include (unless contraindication): receptor blockers, angiotensin transtase inhibitors (ACE-I) or angiotensin receptor blockers (ARB) and ensure individualized optimal drug dose throughout treatment; and aldosterone receptor antagonists; 2) allow the use of other drugs, including oral digitalis, diuretic, hydrazine benzazine, nitrate, warfarin, calcium antagonists, antiplatelets, statins, positive muscle drugs, etc.

**4. Efficacy evaluation and safety evaluation**

Efficacy evaluation indicators: (1) Hemodynamic improvement: USCOM monitoring CI, CO, SVI, SVRI; (2) reduction of BNP;

Safety assessment: (1) Changes in blood pressure, heart rate and respiration before and after treatment; (2) Regular blood routine, liver and renal function, and electrolyte changes before and after treatment. (3) Record and evaluate any adverse event occurring and its correlation with the study drug.

**5. Adverse event was reported**

Any serious adverse event related to or unrelated to the test drug within 30 days of drug administration, must be reported to the principal investigator, the sponsor, the center ethics committee and the local ethics committee within 24 hours.

**6. Stochastic**

The central randomized system was used in this study.Eligible subjects will be randomized to receive Levosimendan + NS or Levosimendan + Shenfu Injection in a 1:1 ratio.

**7. Statistical analysis**

Sample size determination: including the main study indicators, cardiac index, cardiac output, beat output index, peripheral vascular resistance index, BNP, creatinine, urea nitrogen, A LT, AST, calculate the mean and standard deviation, 90% according to the test efficiency, 0.05 for the test level to estimate the sample size. The results showed that n = 96. To avoid the loss in the selected patients, the sample size was n = 100.

**8. Test management and quality control**

**8.1. The Ethical section**

This trial fully followed the Management Code for Drug Clinical Trials and approved the ethical review of our hospital.

**8.2. Quality assurance and audit**

The clinical study was reviewed for quality assurance by persons authorized by our hospital or our hospital. The quality auditor can access all medical records, documents and letters related to the study, as well as informed consent documents for the clinical trial.

**8.3. Informed consent form**

It is the investigator to explain the purpose, methods, benefits, and potential risks of this clinical trial to each subject. Informed consent must be obtained signed by the subject before any operating procedure related to the clinical trial. Informed consent should be expressed, both orally and in writing. Informed consent must be given the date and signed by the subject himself. Signed copies of the informed consent form and the information pages were kept by the subject.

**8.4. Supervise**

Shanghai Tongren Hospital designated inspectors to conduct on-site inspection. The supervisor is from our CRO company authorized by our hospital and needs to operate as the SOP of CRO company. The supervisor should visit regularly from the beginning to the end of the study. Supervisors can access the original data related to this clinical study and review the database as per SOP to determine the integrity, accuracy, and consistency with the original data. Original documents, copies of laboratory data, and medical testing results must be available to clinical supervisors at any time. The supervisor needs to review all the databases and the signed informed consent forms.

**8.5. Confidentiality agreement and subject privacy**

Researchers and stakeholders must maintain the privacy of the clinical trial subjects. In all submitted documents, only the identity of the clinical trial subjects can be identified by the subject number of the clinical trial, but not the name of the subjects and their hospitalization number. The investigator must properly keep the name address of the clinical trial subjects and the entry table corresponding to the subject number.

**8.6. Insurance and indemnity**

In this study, our hospital and the principal investigators should provide insurance for the subjects participating in the clinical study, and bear the cost of treatment and the corresponding financial compensation for the subjects who have suffered the trial-related damage or death.

**9. Estimated progress**

2020-09-01 to 2021-08-31: evaluate cardiac index, cardiac output, output per beat, peripheral vascular resistance index, blood pressure, blood pressure, heart rate, respiration, oxygen saturation, BNP, creatinine, urea nitrogen, A LT, A ST, and relevant data were recorded.

2021-09-01 to 2022-02-29: Organize, analyze and summarize the data.

2022-03-01 to 2020-08-31: publish 1-2 SCI papers, and strive for 1-2 scientific research projects of the Health Commission.
