# Supplementary material for: Use of levosimendan combined with Shenfu injection to treat acute heart failure patients with hypotension: a prospective randomized controlled single-blind study
Source: BMC Cardiovasc Disord. 2022 Mar 29;22:130. doi: 10.1186/s12872-022-02572-2 (PMC8966164; doi:10.1186/s12872-022-02572-2)
Supplement: Supplementary file 2 — Additional file 2: Supplementary Table 1. Medications used during the admission. [file 12872_2022_2572_MOESM2_ESM.docx]

**Supplementary Table 1. Medications used during the admission**

|  | **Levosimendan**  **(N = 51)** | **Levosimendan + SFI**  **(N = 50)** | ***P*-value** |
| --- | --- | --- | --- |
| **Torsemide** |  |  |  |
| N, % | 45 (88.24) | 43 (86.00) | 0.775 |
| Dose (mg, qd), median (IQR) | 20 (20, 20) | 20 (10, 20) | > 0.999 |
| **Tolvaptan** |  |  |  |
| N, % | 6 (11.76) | 5 (10.00) | 1.000 |
| Dose (mg, qd), median (IQR) | 7.5 (7.50, 7.50) | 15 (7.50, 15.00) | 0.242 |
| **Dapagliflozin** |  |  |  |
| N, % | 21 (41.18) | 24 (48.00) | 0.551 |
| Dose (mg, qd) | 10 | 10 | - |
